# Prevalence of Metabolic Abnormalities and Association with Obesity among Saudi College Students

**DOI:** 10.1155/2012/819726

**Published:** 2012-12-19

**Authors:** Mostafa A. Abolfotouh, Ibrahim A. Al-Alwan, Mohammed A. Al-Rowaily

**Affiliations:** ^1^King Abdullah International Medical Research Center (KAIMRC), Riyadh 11426, Saudi Arabia; ^2^King Saud bin-Abdulaziz University for Health Sciences, National Guard Health Affairs, Riyadh 11426, Saudi Arabia

## Abstract

*Aim*. (i) To estimate the prevalence of the metabolic abnormalities among Saudi college students in Riyadh, Saudi Arabia, and (ii) to investigate the association between different indicators of body composition and these abnormalities. *Methods*. A total of 501 college students participated in a cross-sectional study. Anthropometric assessments, BP measurements, and biochemical assessment were done. Metabolic abnormalities were identified. *Results*. Applying BMI, 21.9 % and 20.6% of students were classified as overweight and obese, respectively. Central obesity was prevalent in 26.9% and 42.2% of students based on WC and WHtR, respectively. Other metabolic abnormalities were hypertension (23.6%) and abnormal FPG level (22.6%). Three or more abnormalities were prevalent in 7.8% of students and increased significantly to 26.4%, 20%, and 17.6 in obese subjects based on BMI, WC, and WHtR, respectively. With the exception of abnormal FPG, prevalence of individual metabolic abnormalities as well as the number of these abnormalities significantly increased with increasing BMI, WC, and WHtR (*P* < 0.001 each). *Conclusion*. Our findings provide evidence for the presence of MS in Saudi college students. Central adiposity contributes to the high incidence of individual MS components. College health programs that promote healthful lifestyle and avoidance of adult weight gain are recommended.

## 1. Introduction

An association among obesity, high levels of fasting triglycerides, elevated fasting plasma insulin levels, impaired glucose tolerance, hypertension, and cardiovascular disease (CVD) has been recognized since the early 1960s. These major risk factors tend to cluster in many individuals, suggesting a common etiology that has been variously termed syndrome X, insulin-resistance syndrome, and metabolic syndrome (MS) [1]. Current estimates indicate that the age-adjusted prevalence of metabolic syndrome is roughly 24% among U.S. adults [2]. Although it has been studied extensively in adults, much less is known about metabolic syndrome in youth. Current estimates indicate that roughly 4% of US adolescents have a metabolic syndrome, based on the results of a previous study [3]. A recent study showed a prevalence of 6.8% among the US college-aged students [4].

The prevalence of obesity is increasing substantially, and obesity is one of the major contributors to the incidence of various diseases due to its pathophysiological link to other cardiovascular risks such as hypertension and diabetes. A recent study in Nepal reported that a high incidence of dyslipidemia and abdominal obesity could be a major contributor to MS [5]. Studies from Eastern Mediterranean countries indicate that obesity has reached an alarming level among children and adults. Consequently, the incidence of noncommunicable diseases (NCD) is very high and accounts for more than 50% of total death in the East Mediterranean Region (EMR) [6, 7]. A study of Saudi children and adolescents concluded that obese Saudi children and adolescents have multiple risk factors associated with metabolic syndrome [8]. However, no such association has been studied in Saudi adults. This study aimed to estimate the prevalence of metabolic abnormalities among Saudi college students in Riyadh, Saudi Arabia, and to investigate the association between different indicators of body composition and these abnormalities.

## 2. Materials and Methods

### 2.1. Study Design

A cross-sectional study was conducted with a total of 501 college students aged 18–26 years (383 males and 118 females) from the Colleges of Medicine and Nursing at the King Saud bin-Abdulaziz University for Health Sciences, National Guard Health Affairs, in Riyadh, Saudi Arabia. 

### 2.2. Study Population and Sampling Technique

Students enrolled in the College of Medicine (male students) and the College of Nursing (female students) from 2009 to 2010 were considered the target population of this study.

### 2.3. Techniques

#### 2.3.1. Assessment of Body Composition

All measurements were conducted in college clinics and performed twice by a trained team from March 2010 to August 2010. Body weight was measured using a Seca 634 digital electronic platform scale (Birmingham, UK) with precision to 0.1 kg according to a standardized procedure (lightly dressed, without shoes). Standing height was measured to the nearest 0.1 cm with the use of a stadiometer. BMI was calculated by dividing weight in kg by height squared in meters. Students were classified as follows: underweight (<20 kg/m^2^), normal (20–<25 kg/m^2^), overweight (25–<30 kg/m^2^), and obese (30+ kg/m^2^). WC was measured twice using a nonelastic flexible tape at the smallest abdominal position between the iliac crest and the lower rib margin at the end of normal expiration while the subjects were standing. The measurements were recorded to the nearest 0.5 cm. WHtR was calculated as the ratio of waist (cm) to height (cm) [9]. Blood pressure (BP) was measured twice on the arm with the student seated after rest, using a digital sphygmomanometer and appropriately sized cuff. A fasting blood sample was drawn to determine blood glucose and total cholesterol levels (Vitalab Selectra 2, Merck, Germany). The plasma glucose concentration was determined by the glucose oxidase-peroxidase method. 

#### 2.3.2. Definition of Metabolic Abnormalities

The World Health Organization [10] described MS as the presence of type 2 diabetes or impaired glucose tolerance with any two of the following characteristics: obesity, high levels of triglycerides, low levels of high-density lipoprotein, or hypertension. The International Diabetes Federation (IDF) takes central obesity as a prerequisite for the diagnosis of MS with the association of any two of the other factors listed above [11]. The IDF has derived specific reference values for central obesity for different ethnicities. 

As analyses for elevated TG or reduced HDL-C are not routinely performed for college students (only total blood cholesterol level is estimated), the following metabolic abnormalities were considered in this study: (1) central obesity based on (i) elevated WC ≥ 94 cm (male) or ≥80 cm (female) (these are the European cut-offs recommended by the IDF for use in East Mediterranean and Middle East (Arab) populations until more specific data are available) [12], or (ii) WHtR of 0.5 or more; (2) abnormal fasting plasma glucose (FPG) level (≥5.6 mmol/L); (3) elevated BP of ≥130/85 mm Hg; (4) abnormal total cholesterol level of ≥5.18 mmol/L.

#### 2.3.3. Data Analysis

A statistical analysis was conducted using SPSS (Statistical Package for the Social Sciences) and EPI Info Statistical Package software. Descriptive measures, such as the arithmetic mean and standard deviation, were used to describe the quantitative data. Quartiles of waist circumference and waist-to-height ratio were determined; first, second, and third quartiles were calculated using the frequencies procedure. A computer program placed each WC and WHtR value in one of the following quartile bands: <Q1, <Q2, <Q3, and >Q3.

For the quantitative data, Student's *t*-test was used to compare the sample means. Pearson's chi-squared test was used to compare the categorical data. The chi-squared test for linear trend was used to establish whether the increasing quartiles of WC and WHtR were associated with increased prevalence and number of metabolic abnormalities. Odds ratios were calculated with a 95% confidence interval for the likelihood of a student to have a metabolic abnormality according to the different quartiles. The first quartile groups for WC, WHtR, and BMI of <20 kg/m^2^ were used as the reference categories for each risk variable. Statistical significance was set at *P* < 0.05.

#### 2.3.4. Ethical Consideration

All measurements were performed in private rooms in college clinics. Written informed consent was obtained from students before any testing procedure. All participants had the right not to participate in the study or to withdraw from the measurements prior to completion. The study protocol received ethical approval from the Institutional Review Board of King Abdullah International Medical Research Center (KAIMRC), King Saud bin-Abdulaziz University for Health Sciences, Riyadh, Saudi Arabia (Ref no. RR08/063).

## 3. Results and Discussion

The prevalence of obesity is increasing substantially, and obesity is one of the major contributors to the incidence of various diseases due to its pathophysiological link to other cardiovascular risks such as hypertension and diabetes [7].  Table 1 shows the distribution of study samples according to body composition measures and metabolic abnormalities. The prevalence of overweight and obesity based on BMI was 42%. Central obesity was prevalent in 26.9% of the study sample and was prevalent in 42.2% of the sample when using WC and WHtR measures.

Central obesity was the most common metabolic abnormality among Saudi students, followed by high systolic blood pressure (36%), high systolic and/or diastolic blood pressure (23.6%), and abnormal fasting plasma glucose levels (22.6%). This finding was in agreement with the finding of a previous study of adult Saudi soldiers [13]. However, this study's findings contradict the finding that a high fasting glucose level was the least common factor among US adolescents [1]. This disagreement can be attributed to the higher cutoff point used for abnormal fasting glucose levels in the US study. Other metabolic abnormalities included high diastolic blood pressure (8.6%) and abnormal total cholesterol levels (9.6%). These findings were similar to the results for adults in Nepal [7] and Ethiopia [14]. Males showed significantly higher prevalence of the following abnormalities: high SBP (*χ* ^2^ = 38.31, df = 1, *P* < 0.001), high DBP (*χ* ^2^ = 8.65, df = 1, *P* = 0.003) and high SBP and DBP (*χ* ^2^ = 38.37, df = 1, *P* < 0.001) obesity based on BMI (44.7% versus 35.6%, *χ* ^2^ = 9.21, df = 3, *P* = 0.027); WHtR indicators (46.6% versus 32.5%, *χ* ^2^ = 6.58, df = 1, *P* = 0.01).

**Figure 1:**
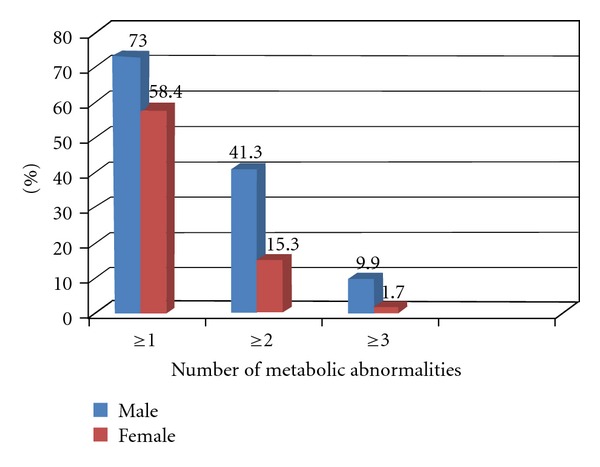
Prevalence of the ≥1, ≥2, and ≥3 metabolic abnormalities among Saudi college students by sex.

**Figure 2:**
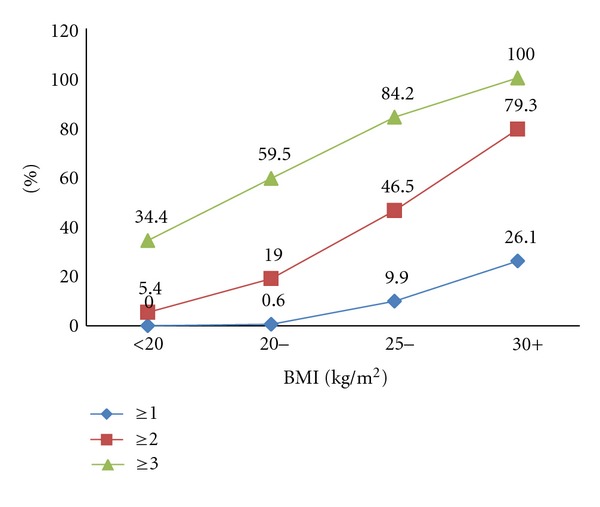
Prevalence of the ≥1, ≥2, and ≥3 metabolic abnormalities among Saudi college students by BMI levels.

The proportion of students with one or more metabolic abnormalities is presented in Figure 1. Approximately 68% of students had one or more abnormalities and 34.5% had two or more abnormalities. These values are higher than those observed among US adolescents (43% and 17% for ≥1 and ≥2 abnormalities, resp.) [1]. Students with three or more abnormalities were 7.7%. Also, this figure was slightly higher than its corresponding figure of 6.8% among the US college-aged students in a recently published study [4]. This difference can be attributed to sedentary lifestyle and dietary habits, which are important predictors of obesity and metabolic abnormalities [15, 16], However, a study in Nepal showed no significant difference in the prevalence of MS between those who did not engage in physical activity and those that did engage in physical activity [7]. Thus, further studies are needed to investigate this association. Males constituted a significantly higher proportion of subjects with three or more abnormalities, which is in agreement with other studies [7, 17–19].  Figure 2 shows a significant increase in the proportions of students with ≥1, ≥2, and ≥3 metabolic abnormalities with increases in the BMI.

**Figure 3:**
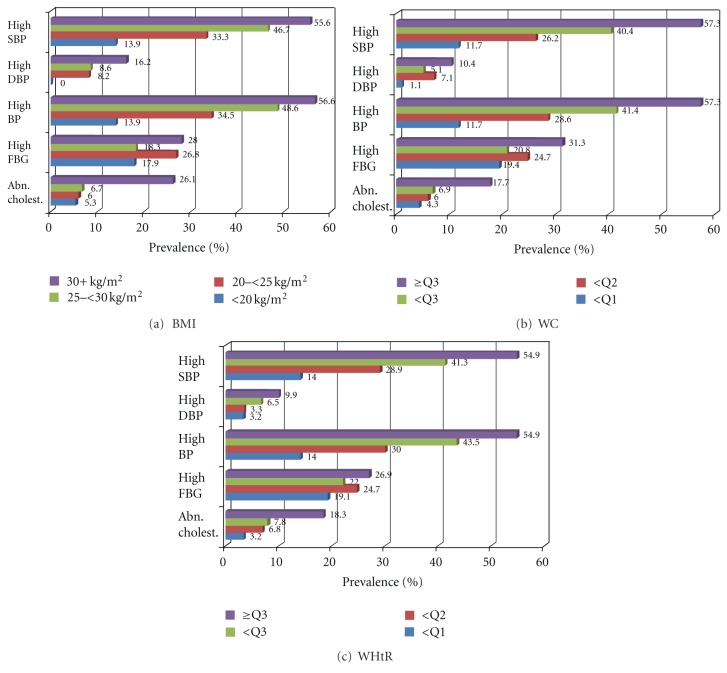
Prevalence of individual metabolic syndrome abnormalities in relation to (a) BMI, (b) WC, and (c) WHtR obesity indicators among Saudi college students.

BMI is a widely used general obesity index. In a US study, metabolic syndrome was largely confined to overweight and obese adults, with prevalence estimates of approximately 22% among adults with a BMI ≥ 25 and <30 kg/m^2^ and 60% among adults with a BMI ≥ 30 kg/m^2^[20]. In the present study, there was a significant increase in the prevalence of 3 or more metabolic abnormalities with increasing BMI (*χ*_LT_^2^ = 54.01, *P* < 0.001). The odds ratio for a BMI of 30+ kg/m^2^ versus a BMI of <20 kg/m^2^ was 4.5 (95% CI: 1.9–10.8). Moreover, there was an appreciable increase in the risk of high SBP with increasing BMI (*χ*_LT_^2^ = 43.12, *P* < 0.001). The odds ratio for a BMI of 30+ kg/m^2^ versus a BMI of <20 kg/m^2^ was 7.9 (95% CI: 1.9–10.8) (Table 2; Figure 3(a)). This finding was the same for high DBP, high overall BP, and abnormal cholesterol levels, each significantly increased with increasing BMI level. However, in a recent study by Dalleck and Kjelland [4], BMI was not independently associated with any MS component after partialling out the effect of physical activity. This was in agreement with a previous study of the Egyptian adults, where the BMI per se was not a significant predictor for diabetes or hypertension after adjustment for other factors such as age, sex, and smoking behavior [21]. However, in the present study, there was no significant increased risk of high fasting plasma glucose with an increase in any of these body composition measures. This finding can be explained by the hypothesis that glucose intolerance may develop later than other syndrome abnormalities [22], especially in a group of young adults, such as the college students (age 26 or younger) in the present study.

Central adiposity appears to contribute to the development of cardiovascular risk to a greater extent than general adiposity does [23]. Central adiposity is an important indicator of cardiovascular disease due to its link to dyslipidemia, hyperinsulinemia, hypertension, and impaired fibrinolytic capacity [24]. WC and WHtR are also important indicators of central obesity [25, 26]. In this study, there was a significant increase in the prevalence of 3 or more metabolic abnormalities with increasing WC quartile (*χ*_LT_^2^ = 33.85, *P* < 0.001) and with increasing WHtR quartile (*χ*_LT_^2^ = 24.344, *P* < 0.001). The odds ratio for >Q3 of WC versus <Q1 was 2.4 (95% CI: 1.2–6.6), and the odds ratio for >Q3 of WHtR versus <Q1 was 3.6 (Table 2; Figures 3(b) and 3(c)). Moreover, there was an appreciable increase in the risk of high SBP with increasing WC quartile (*χ*_LT_^2^ = 47.69, *P* < 0.001) and with increasing WHtR quartile (*χ*_LT_^2^ = 37.05, *P* < 0.001). The odds ratio for >Q3 of WC versus <Q1 was 10.12 (95% CI: 1.2–6.6), and the odds ratio for >Q3 of WHtR versus <Q1 was 7.5. This significant association between abdominal obesity and MS abnormalities is because both MS and increased abdominal fat are related to a reduction of adiponectin, an adipocyte-derived hormone with antiatherogenic and anti-inflammatory properties [27]. Once again, this finding was the same for all other metabolic abnormalities except the fasting plasma glucose level. In a recent study of US college-aged students, WC was independently associated with three of the MS components (HLD-C, TG, and SBP) after partialling out the effect of physical activity [4]. In a previous study among Saudi geriatrics, WC was a powerful independent predictor only of hypertension risk, while WHC was a good predictor only of diabetes risk [28].

**Figure 4:**
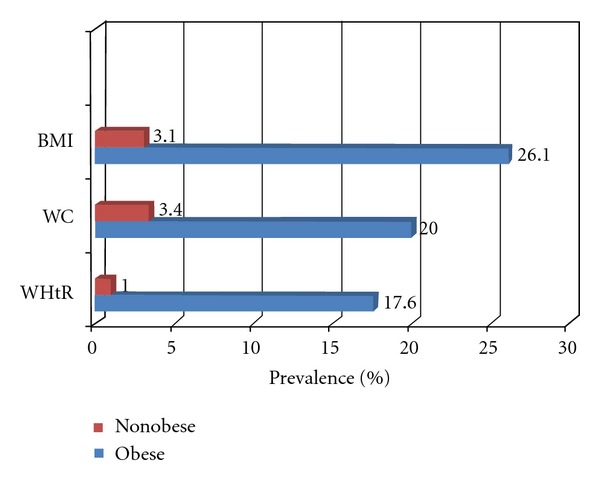
Prevalence of 3 or more metabolic syndrome abnormalities in obese and nonobese college students.

It has been reported that WC was better than BMI as a predictor of CVD risk factors and metabolic abnormalities [29–31]. In this study, assuming students with 3 or more abnormalities had MS, the prevalence of MS was 7.8%. This prevalence increased from 3.1% in nonobese to 26.1% among obese students based on BMI, from 3.4% to 20% based on WC, and from 1% to 17.7% based on WHtR measures (Figure 4). This finding showed that BMI, WC, and WHtR were equally predictive of CVD risk. This result is consistent with the results from a 2007 meta-analysis [32] that suggested that measures of overall obesity (BMI) and measures of central obesity (WHR and WC) performed equally well in predicting the incidence of type 2 diabetes. WHtR was suggested as the best indicator of hypertension, diabetes and dyslipidemia by some investigators [33–35], while WC was suggested as the best for the prediction of diabetes and hypertension [21]. However, this study found that fasting plasma glucose levels were not significantly associated with any of these obesity indicators. This disagreement can be attributed to different lifestyle factors [36]. Ethnic differences in body composition rules and constants are also important, especially in relation to being overweight or obese [37].

This study has some limitations, which should be considered when interpreting these findings. First, the study used a cross-sectional design that did not allow for the assessment of the temporality of the relationship between body composition measures and metabolic abnormalities, especially that other confounding factors such as physical activity and dietary habits were not adjusted for proving this association. A longitudinal study with repeated measures of body composition and metabolic abnormalities would be desirable in the future. Second, measurements of HDL-C, TG, hormone, and biomarker levels, which are known to be involved in the pathogenesis and identification of MS in Saudi college students, were not included in the present study. These excluded measurements may have led to an underestimation of the prevalence of MS abnormalities. However, aside from this possible underestimation, the validity of the conclusion with regard to study's individual parameters is preserved.

The overall results of our study provide an evidence for the presence of MS components among Saudi college students. Measures of adiposity are correlated with metabolic abnormalities among Saudi students. BMI, WC, and WHtR were all suitable indicators that could be used to predict metabolic parameters. Although this association has already been studied in the literature, yet the data of this study is instructional given the understudied young Saudi population. The prevalence of metabolic abnormalities and obesity and the observed association of adiposity with these abnormalities calls for increased efforts towards clinical preventive services to identify and control existing metabolic abnormalities among students. Additionally, the development and implementation of college health programs that promote healthful behaviors, including increased physical activity, eating balanced diets, and avoidance of adult weight gain, are needed to help reduce the burden of noncommunicable diseases among Saudi students and the broader Saudi community. 

## Figures and Tables

**Table 1:** Body composition and metabolic abnormalities among 501 Saudi college students aged 18–26 years.

	Male		Female		Total	
	No.	%	No.	%	No.	%
BMI						
<20	68	(18.1)	36	(30.5)	104	(21.1)
20–<25	140	(37.2)	40	(33.9)	180	(36.4)
25–<30	84	(22.3)	24	(20.3)	108	(21.9)
30+	84	(22.4)	18	(15.3)	102	(20.6)

Total	376	100.0	118	100.0	494	100.0
Sex difference	*χ* ^2^ = 9.207, df = 3, (*P* = 0.027)					

Waist circumference, cm						
Normal	195	(73.3)	85	(72.6)	280	(73.1)
Obese	71	(26.7)	32	(27.4)	103	(26.9)

Total	266	100.0	117	100.0	383	100.0
Sex difference	*χ* ^2^ = 0.018, df = 1, (*P* = 0.089)					

Waist-to-height ratio						
Normal	140	(53.4)	79	(67.5)	219	(57.8)
Obese	122	(46.6)	38	(32.5)	160	(42.2)

Total	262	100.0	117	100.0	379	100.0
Sex difference	*χ* ^2^ = 6.58, df = 1, (*P* = 0.01)					

Systolic BP, mm Hg						
Normal	214	(55.9)	103	(87.3)	317	(63.3)
High	169	(44.1)	15	(12.7)	184	(36.7)

Total	383	100.0	118	100.0	501	100.0
Sex difference	*χ* ^2^ = 38.31, df = 1, (*P* < 0.001)					

Diastolic BP, mm Hg						
Normal	344	(89.8)	116	(98.3)	460	(91.8)
High	39	(10.2)	2	(1.7)	41	(8.2)

Total	383	100.0	118	100.0	501	100.0
Sex difference	*χ* ^2^ = 8.65, df = 1, (*P* = 0.003)					

High BP						
Normal	210	(54.8)	102	(86.4)	383	76.4
High	173	(45.2)	16	(13.6)	118	23.6

Total	383	100.0	118	100.0	501	100.0
Sex difference	*χ* ^2^ = 38.367, df = 1, (*P* < 0.001)					

Fasting blood glucose, mmol/L						
Normal	274	(75.3)	99	(83.9)	373	(77.4)
High	90	(24.7)	19	(16.1)	109	(22.6)

Total	364	100.0	118	100.0	482	100.0
Sex difference *χ* ^2^, *P* value	*χ* ^2^ = 3.79, df = 1, (*P* = 0.052)					

Cholesterol level						
Normal	321	(88.9)	112	(94.9)	433	(90.4)
Borderline	33	(9.1)	5	(4.2)	38	(7.9)
High	7	(2.0)	1	(0.9)	8	(1.7)

Total	361	100.0	118	100.0	479	100.0
Sex difference	*χ* ^2^ = 3.684, df = 2, (*P* = 0.158)					

**Table 2:** Prevalence of individual metabolic abnormalities and the association between these abnormalities and different indicators of obesity among 501 Saudi college students aged 18–26 years.

	High SBP			High DBP			Overall HBP			High fasting blood glucose levels			Abnormal cholesterol levels			3+ risk factors		
	Prev.	(*n*, %)	OR	Prev.	(*n*, %)	OR	Prev.	(*n*, %)	OR	Prev.	(*n*, %)	OR	Prev.	(*n*, %)	OR	Prev.	(*n*, %)	OR
BMI levels (kg/m^2^)																		
<20	14	(13.9)	1^@^	0	(—)	1^@^	14	(13.9)	1^@^	17	(17.9)	1^@^	5	(5.3)	1^@^	0	(—)	1^@^
20–<25	57	(33.3)	3.11	14	(8.2)	9.01	59	(34.5)	3.27	45	(26.8)	1.68	10	(6.0)	1.15	1	(0.6)	0.57
25–<30	49	(46.7)	5.33	9	(8.6)	9.57	51	(48.6)	5.75	19	(18.3)	1.04	7	(6.7)	1.31	10	(9.8)	10.22
30+	55	(55.6)	7.91	16	(16.2)	19.24	56	(56.6)	8.24	26	(28.0)	1.75	24	(26.1)	6.26	24	(26.4)	32.82
*χ*_LT_^2^, *P* value	43.12, (*P* < 0.001)			15.316, (*P* < 0.001)			43.34, (*P* < 0.001)			0.855, (*P* < 0.36)			21.33, (*P* < 0.001)			54.01, (*P* < 0.001)		

WC quartiles																		
<Q1	11	(11.7)	1^@^	1	(1.1)	1^@^	11	(11.7)	1^@^	18	(19.4)	1^@^	4	(4.3)	1^@^	0	(—)	1^@^
<Q2	22	(26.2)	2.68	6	(7.1)	7.15	24	(28.6)	3.02	21	(24.7)	1.39	5	(6.0)	1.43	1	(1.2)	1.15
<Q3	40	(40.4)	5.12	5	(5.1)	4.95	41	(41.4)	5.33	21	(20.8)	1.11	7	(6.9)	1.66	7	(7.2)	7.23
≥Q3	55	(57.3)	10.12	10	(10.4)	10.81	55	(57.3)	10.12	30	(31.3)	1.92	17	(17.7)	4.79	21	(22.6)	27.12
*χ*_LT_^2^, *P* value	47.69, (*P* < 0.001)			5.824, (*P* = 0.016)			46.405, (*P* < 0.0001)			2.6, (*P* = 0.106)			9.79, (*P* = 0.002)			33.85, (*P* < 0.001)		

WHtR quartiles																		
<Q1	13	(14.0)	1^@^	3	(3.2)	1^@^	13	(14.0)	1^@^	18	(19.1)	1^@^	3	(3.2)	1^@^	1	(1.1)	1^@^
<Q2	26	(28.9)	2.50	3	(3.3)	1.03	27	(30.0)	2.64	22	(24.7)	1.39	6	(6.8)	2.22	1	(1.1)	1.07
<Q3	38	(41.3)	4.33	6	(6.5)	2.09	40	(43.5)	4.73	20	(22.0)	1.19	7	(7.8)	2.56	9	(10.0)	10.48
≥Q3	50	(54.9)	7.50	9	(9.9)	3.29	50	(54.9)	7.50	25	(26.9)	1.55	17	(18.3)	6.79	17	(18.9)	21.42
*χ*_LT_^2^, *P* value	37.05, (*P* < 0.001)			4.521, (*P* < 0.033)			37.267, (*P* < 0.000)			1.112, (*P* < 0.292)			12.099, (*P* < 0.001)			24.344, (*P* < 0.000)		

^@^reference categories for each risk variable.

## Abbreviations

BMI: body mass index
WC: waist circumference
WHtR: waist to height ratio
SBP: systolic blood pressure
FPG: fasting plasma glucose.
